# Sociocultural Influences, Drive for Thinness, Drive for Muscularity, and Body Dissatisfaction among Korean Undergraduates

**DOI:** 10.3390/ijerph17145260

**Published:** 2020-07-21

**Authors:** Sukkyung You, Kyulee Shin

**Affiliations:** 1 College of Education, Hankuk University of Foreign Studies, Seoul 130-791, Korea; skyou@hufs.ac.kr; 2 Department of Sports Sciences, Seoul National University of Science & Technology, Seoul 01811, Korea

**Keywords:** sociocultural influences, drive for thinness, drive for muscularity, body dissatisfaction, exercise

## Abstract

For many years, body dissatisfaction was considered a western phenomenon, and was studied mostly in Caucasian women. Recent studies, however, suggest that these issues are also present in men and in other ethnic groups. This research investigated the differential effects of various sociocultural pressures transmitted from the media, one’s parents, and one’s peers on the drives for thinness and muscularity, and body dissatisfaction among 1125 Korean college students (56% male) using structural equation modeling. The results indicate that, after controlling for body mass index and exercise, media pressures exerted the largest effects on participants’ body ideals and, in turn, body dissatisfaction across both genders (*β* = 0.44, and 0.30, *p* < 0.05, for females and males, respectively). This study’s results also indicate that there are considerable gender differences in this relationship. Specifically, the results show that parental and media pressure had significant indirect relationships with body dissatisfaction via the drive for thinness among females, while peer and media pressures had significant indirect relationships with body dissatisfaction via the drive for muscularity among males. As body dissatisfaction is known to significantly affect an individual’s mental and physical health, future research needs to identify relevant influential factors in this area, as well as the paths they have leading to increased body dissatisfaction.

## 1. Introduction

Body dissatisfaction has primarily been studied among females under the assumption that men were unlikely to suffer from serious problems in this regard. However, recent studies suggest that it is a problem for males, with male body concerns differing from female ones [1,2,3]. In other words, females tend to desire thinner bodies, while males tend to desire more muscular physiques. Additionally, recent evidence suggests that current Western body ideals are shifting toward toned, thin physiques for women and lean, muscular ones for men [4]. Thus, young adults’ body image disturbances are a complex matter; therefore, a need exists to understand body ideals and their negative consequences. Hence, the main goal of this study is to examine the effects of sociocultural pressures on both drives for thinness and muscularity, and body dissatisfaction among both genders.

### Sociocultural Influences on the Body Ideal

Both men and women have become increasingly dissatisfied with their body types, perhaps because of unrealistic societal standards around physical attractiveness. A drive for thinness is associated with negative behavioral and/or psychological problems such as disordered eating, depression, lower self-esteem, and body dissatisfaction [5]. Similarly, a drive for muscularity is often detrimental to one’s health and body image [3].

To understand the emergence of body dissatisfaction, the tripartite influence model of body image [6] proposes that social pressures from the media, one’s parents, and one’s peers lead to body dissatisfaction via societal body ideal internalization. This model has been extended and has received support in various studies conducted in adult and adolescent samples. Tylka [7] refined the tripartite influence model by including dual body image pathways (i.e., drive for thinness and muscularity). Previous studies indicate that the media has the greatest influence on peoples’ body images. Its influence is a strong predictor for the drive for thinness and body dissatisfaction among females and males [1,8,9]. Although, notably, men may not only want to lose weight, they also eventually want to gain muscle [10]. Studies have found that media pressures influence both the drives for thinness and muscularity among males, causing disordered eating among many adolescent boys [1,2] and men [11]. This implies the existence of gender differences in sociocultural standards of body ideals. Eating disorders are occurring very frequently among adolescents and young adults, and nearly 1 out of 10 individuals with disordered eating behaviors are men, indicating that disordered eating behaviors can occur regardless of gender [12].

Parents are also significant social factors in the development and maintenance of body image. Research has found that parental comments on weight loss predict disordered eating behaviors and negative body images among adolescent girls [13], female undergraduates [14], and adolescent boys [15].

Additionally, a person’s peers are another important sociocultural component. Peer comments have a powerful influence on harmful body-related behaviors and dissatisfaction [16]. A study among female undergraduates found that negative body-related talk between friends was positively associated with body dissatisfaction [17]. Likewise, previous research found that friends’ teasing of people’s weight and physique significantly influences male adolescents’ obsessive muscle building [18], muscularity internalization, and body dissatisfaction [19]. In Valois et al.’s [20] longitudinal study, weight teasing by one’s peers was associated with subsequent body dissatisfaction over two years among adolescent boys and girls.

Furthermore, research on body image and dissatisfaction among men of ethnic minorities remains limited, with previous studies focusing on White men’s experiences [21]. Some studies have found that Asian/Asian American men in the U.S. felt smaller than their body ideals, indicating a desire for increased muscularity [22]. Asian American undergraduate men in the U.S. reported higher drives for muscularity and body dissatisfaction, with greater media internalization of body ideals compared to White and Black males [23]. Additionally, a study among young Taiwanese males discovered that increased muscularity pursuits are associated with body dissatisfaction and exercise dependence [24]. 

Given ethnicity’s significant role in the relationship between sociocultural pressures, drive for body ideals, and body dissatisfaction, this study investigated the relationships between these factors using a sample of Korean college students to provide a culturally appropriate framework to understand them. Furthermore, past research has found that possessing both thinness and muscularity drives tends to be associated with negative psychological and behavioral outcomes [25]. Therefore, this study specified distinct types of sociocultural pressures to provide a more detailed understanding of the processes underlying each one’s relationship with the drives for thinness and muscularity among both genders. Moreover, this study examined whether the drives for thinness and muscularity are mediators between sociocultural pressures and body dissatisfaction.

## 2. Materials and Methods 

### 2.1. Sample

The study participants were college students recruited from three private universities in Seoul, Korea. Of the surveys distributed, 96.1% were completed and returned. In total, 1125 students provided usable surveys (56% male). The sample’s mean age was 23.52 years (SD = 5.60 years).

### 2.2. Procedures

After receipt of institutional review board approval, data were collected during the Fall semester, 2019. Instructors from the College of Health Sciences were contacted to obtain permission to recruit the study’s participants from their classes. Full-time academic students majoring in Health Sciences were recruited. A majority of them were taking 15 to 18 academic credits per semester. Once approval was granted, trained researchers distributed an information letter, describing the study. All subjects gave their informed consent for inclusion before they participated in the study. Those who opted to participate were administered the survey, in paper and pencil format, during class. The study was conducted in accordance with the Declaration of Helsinki, and the entire survey was reviewed and approved by a professional panel of educational specialists and school counselors (Project identification code No. CSED-2019-001A).

### 2.3. Measures

#### 2.3.1. Drive for Thinness

The Drive for Thinness subscale of the Eating Disorder Inventory (EDI-DFTS; [26]) was used to measure participants’ drive for thinness. The scale was used to assess participants’ preoccupation with body weight, excessive dieting concerns, entrenchment in an extreme pursuit of thinness, and any intense fears of becoming fat. It consists of seven items, each instructing respondents to rate their answers from 1 (never true of me) to 6 (always true of me). This scale is often used in Korean body image research and the scale has shown significant validity and reliability (Cronbach’s α range = 0.86–0.91 [27,28]). 

#### 2.3.2. Drive for Muscularity

The Drive for Muscularity Scale (DMS; [29]) was used to measure participants’ preoccupations with becoming more muscular, obsession with muscle building exercise, level of impact of exercise on life, and guilt over skipping exercise. The scale consists of 15 items, each instructing respondents to rate their answers from 1 (never true of me) to 6 (always true of me). This scale is often used in Korean body image research and the scale has shown significant validity and reliability (Cronbach’s α = 0.84 [28]).

#### 2.3.3. Body Dissatisfaction

The Body Dissatisfaction subscale of the Eating Disorder Inventory (EDI-BD; [26]) was used to measure participants’ body dissatisfaction. The scale consists of nine items, each instructing respondents to rate their answers from 1 (never true of me) to 6 (always true of me). The EDI-BD used in this study has been used widely in extant research in Korea, and other research findings have shown that there are significant associations with related variables, such as body consciousness in college students (Cronbach’s α = 0.85 [30]) and eating disorders [27], providing evidence supporting the validity of the instrument for use with this population.

#### 2.3.4. Sociocultural Influences on Appearance

To measure relevant sociocultural influences, 43 items (10 for media, 20 for parental, and 13 for peer influences) of the Tripartite Influence Scale [31] were used. The validity and reliability of each subscale (media, parent, peer pressure) were also confirmed through several studies (Cronbach’s α range = 0.85–0.90 [32,33,34]).

#### 2.3.5. Exercise Behaviors

The Leisure-Time Exercise Questionnaire (LTEQ) is a self-report instrument assessing the frequency of mild, moderate, and strenuous exercise conducted for 15 min or longer during a typical week [35]. The weekly frequencies of mild, moderate, and strenuous exercises are converted into a measure of energy expenditure called “metabolic equivalents” using the following formula: (3 × mild) + (5 × moderate) + (9 × strenuous). The LTEQ is a reliable and valid measure of exercise behavior in adults [36,37].

### 2.4. Analysis

**Figure 1:**
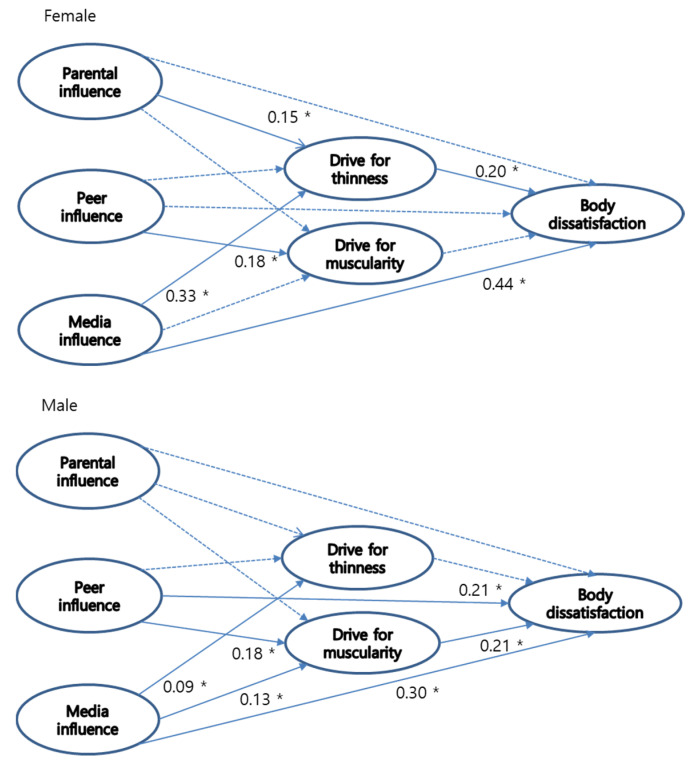
Final model estimation with standardized coefficients. Note. * *p* < 0.05; significant path coefficients are shown in bold line; dotted line = non-significant.

Structural Equation Modeling (SEM) was used to assess the hypothesized structural relationships between the latent variables. SEM was selected because it is an appropriate analytical approach specifying directionality for the variables of interest, while also generating the flexibility with which to test causal relationships. In stage one, a confirmatory factor analysis was conducted to test the measurement model for the tripartite influence model of body image. The fit indices indicated that the three-factor model yielded a good fit to the current sample in terms of the Comparative Fit Index (CFI), NNFI (both over 0.95) and root mean square error of approximation (RMSEA) (.05). In the second stage, a structural model (see Figure 1) tested the hypotheses in the present study. For SEM analysis, we used the questionnaire items as measured variables to represent the latent variables in the model shown in Figure 1. Two mediational models were tested to compare and derive the best one. The model’s fit was assessed, based on several criteria: the Comparative Fit Index (CFI; [38]), non-normed fit index (NNFI; [39]), and the root mean square error of approximation (RMSEA; [40]). Values lower than 0.06 for the RMSEA, and values close to 0.95 for the NNFI and CFI, were used to determine a good-fit model. All analyses were conducted using AMOS [41].

## 3. Results

### 3.1. Sample Descriptive Statistics

The variables’ correlations, means, and standard deviations are provided in Table 1. 

Significant correlations were found among these variables. In examining mean level differences, the results showed significant gender differences for these variables. Specifically, college-going women reported a higher drive for thinness, and a lower drive for muscularity, when compared to their male counterparts. According to the guidelines of severe non-normality (i.e., skew > 2; kurtosis > 7) proposed by West, Finch, and Curran [42], the normality assumption for all the variables was met. 

### 3.2. Testing the Mediational Models

For the male group, the partial mediational model yielded an overall χ^2^(64) value of 420.49, with CFI = 0.967, NNFI = 0.962, and RMSEA = 0.064 and the full mediational model yielding an overall χ^2^(67) value of 483.65, with CFI = 0.844, NNFI = 0.856, and RMSEA = 0.099. For the female group, the partial mediational model yielded an overall χ^2^(64) value of 265.19, with CFI = 0.950, NNFI = 0.934, and RMSEA = 0.060 and the full mediational model yielding an overall χ^2^(67) value of 365.46, with CFI = 0.853, NNFI = 0.866, and RMSEA = 0.080.

A chi-square difference test was conducted that supported the partial mediational model for both the male and female groups. Thus, we chose the partial mediational model as the final theoretical model for both genders. The fit of the final model was deemed acceptable in terms of three fit indices. The standardized parameter estimates for this model are presented in Figure 1.

These results indicate that media pressures have significant effects on the drive for thinness and body dissatisfaction among female undergraduate students, while peer and media pressures have significant effects on the drive for muscularity and body dissatisfaction for male ones. In regards to the mediating effects, the bootstrap test results indicate that parental and media pressures on body dissatisfaction for females (*β* = 0.30, and *β* = 0.07, *p* < 0.05, respectively) via drive for thinness, and peer and media pressures on body dissatisfaction for males (*β* = 0.04, and *β* = 0.03, *p* < 0.05, respectively) via drive for muscularity, were all significant. 

## 4. Discussion

This study aimed to extend the literature on body image by examining the relationship between each domain of sociocultural pressure (media, parents, and peers) and body dissatisfaction, as well as the mediating role of ideal body figure internalization within these relationships, using a non-Western sample. Most research on body dissatisfaction examining the effects of sociocultural pressures on this variable among women assessed media pressure only or combined all sources to measure its social influences. Furthermore, recent studies have found that internalization of two body ideal dimensions—thinness and muscularity—is associated with peoples’ body dissatisfaction. The tripartite influence model includes a test of direct (sociocultural pressures) and mediational links (internalization of societal body ideal standards) as factors that contribute to body dissatisfaction. This model has been extended by including dual body image pathways (i.e., drive for thinness and muscularity) and has received support in an adult male sample [7]. 

Our study findings support Tylka’s refined model of the tripartite influence of body image and dissatisfaction among a sample of Korean college male and female students. Our results found considerable gender differences in these variables. The drives for thinness and muscularity were correlated with all three societal influences across gender groups, but, when the multivariate analyses were conducted, the relative effects of these sources remained different across the genders. 

For the female group, parental and media pressures exerted significant influences on their drive for thinness and, in turn, their body dissatisfaction. Additionally, media pressures were found to have direct influences on their body dissatisfaction, thus lending support to the significant relationships between thin ideal internalization and body dissatisfaction. These findings are consistent with previous studies. Researchers have found that parental influences were positively associated with the drive for thinness among undergraduate females [43] and adolescent girls [44]. Other studies found that the media’s influence had a significant relationship with the drive for thinness among adolescent girls [45], Japanese undergraduates [46], and Australian and French female undergraduates [47]. Additionally, our findings indicate that peer pressure significantly influences the pursuit of muscularity, meaning that, besides thinness, muscle tone is a focus for young Korean women—yet this pursuit does not lead to body dissatisfaction. However, few studies have examined muscularity-related body dissatisfaction among women [48]. Our findings are in line with those of previous studies in that, when we considered the internalization of thinness and muscularity, only that of thin ideals predicted female students’ body dissatisfaction [49,50]. This suggests that, currently, young Korean women feel more insecure about being thin rather than about gaining muscularity, with becoming ultra-thin being of greater focus for their self-esteem. However, a recent Western study found that sociocultural influences (i.e., the media and one’s peers) towards thinness and muscularity idealizations contributed to increased body dissatisfaction and risky body-changing behaviors among young French women [51]. Similar findings indicate that thinness and muscularity idealizations contributed to eating disorders and substance use among German women [52]. 

For the male group, consistent with the findings of extant research conducted in Europe or with white men, the pursuit of the muscular ideal was found to be a vulnerability factor for body dissatisfaction [53]. Specifically, peer and media influences were significantly associated with an increased drive for muscularity and, in turn, body dissatisfaction. Additionally, both peer and media pressures were discovered to have direct influences on men’s body dissatisfaction. This is in line with the findings of previous studies. One study found that social media pressure directly influences both the drive for muscularity and body dissatisfaction among young Korean males [54]. Another study found that appearance-related talk with one’s peers had the largest effect, followed by media internalization, on the drive for muscularity and body dissatisfaction among Korean adolescent boys [55]. That is, when men pursue media ideals as personal goals for their physicality, they are more susceptible to experiencing an increased desire to obtain a more muscular image. In addition, the current findings indicate that media pressures exerted significant influences on men’s drive for thinness. A previous study found that media influences predicted thin-internalization among Hungarian boys [9], indicating that, besides muscularity, slenderness is also a focus for males.

Our findings indicate that the degree of internalization due to mass media pressure is greater than that due to influences from one’s parents and peers, confirming the immense influence that the media has on the internalization of the thinness ideal for women, and that of lean, muscular figures for men, which may further develop into instances of body dissatisfaction. Thus, there is a need for interventions educating young adults in an increased awareness of the media influences on ideal body internalization and body dissatisfaction, as well as on critically evaluating these influences to be more realistic about their own bodies and perceptions around diverse body figures. 

Our findings also showed that internalizing the thin and muscular ideals were independently associated with higher levels of body dissatisfaction; however, the patterns differed across gender groups. Current findings therefore aid health professionals and researchers in better understanding these associations. In many college environments, college health professionals and counselors are actively involved in dealing with body dissatisfaction in female students. However, the current study findings showed that male students also have body image concerns (i.e., internalization of lean, muscular figures) but different ones than females. Thus, more emphasis needs to be given to male students’ body dissatisfaction. For example, college health care professionals need to be aware of the signs of anabolic androgenic steroid use and other types of performance-enhancing supplements and should provide information about the adverse health consequences of steroid abuse as well as the healthy eating practices available for male students. 

This study has certain strengths, including an adequate sample size for the analyses used, and the use of a culture- and gender-specific sample. Furthermore, including different sources of sociocultural support and examining the mediating effects of both the drive for thinness and muscularity addressed the limitations of previous body image studies. However, there are limitations that offer avenues for future research. First, this cross-sectional study predicts relationships between the relevant variables, but does not outline any causality. Future research should thus take a longitudinal approach to examine the causality between these variables. Second, using convenience sampling did not allow for the generalization of the study findings, leaving the need for the inclusion and examination of more diverse groups open.

## 5. Conclusions

Using a Korean undergraduate sample, this study examined the relationships between sociocultural pressures, their internalization, and body dissatisfaction. The results partially support the idea of a significant relationship between sociocultural pressures with body dissatisfaction across both genders. Specifically, among Korean female undergraduates, the drive for thinness was found to mediate the relationships between parental and media pressures and body dissatisfaction; while, among Korean male undergraduates, the drive for muscularity was found to mediate the relationships between peer and media pressures and body dissatisfaction. As body dissatisfaction is known to significantly affect an individual’s mental and physical health, future research needs to identify relevant influential factors in this area, as well as the paths they have leading to increased body dissatisfaction.

## Figures and Tables

**Table 1:** Correlations and descriptive statistics for study variables.

	1	2	3	4	5	6
1. Parent influence **^α^**	1	0.32 *	0.37 *	0.14 *	0.11 *	0.25 *
2. Peer influence **^α^**	0.44 *	1	0.40 *	0.25 *	0.16 *	0.19 *
3. Media influence **^α^**	0.34 *	0.36 *	1	0.23 *	0.28 *	0.23 *
4. Drive for thinness **^α^**	0.10 *	0.09 *	0.07	1	0.32 *	0.21 *
5. Drive for muscularity **^α^**	0.16 *	0.25 *	0.29 *	0.30 *	1	0.07
6. Body dissatisfaction **^α^**	0.18 *	0.21 *	0.18 *	0.04	0.15 *	1
Means *±* SD						
Male	1.61 (0.65)	2.23 (0.86)	2.01 (0.75)	2.70 (1.21)	3.40 (1.03)	2.80 (0.64)
Female	1.82 (0.72)	3.11 (0.84)	2.63 (0.77)	3.83 (1.03)	2.69 (1.01)	3.22 (0.69)
Cronbach’s alphas						
Male	0.79	0.78	0.78	0.79	0.81	0.79
Female	0.82	0.81	0.83	0.83	0.77	0.82

Note. Correlations for females are above diagonal; **^α^** Gender difference is significant at * *p* < 0.05.
